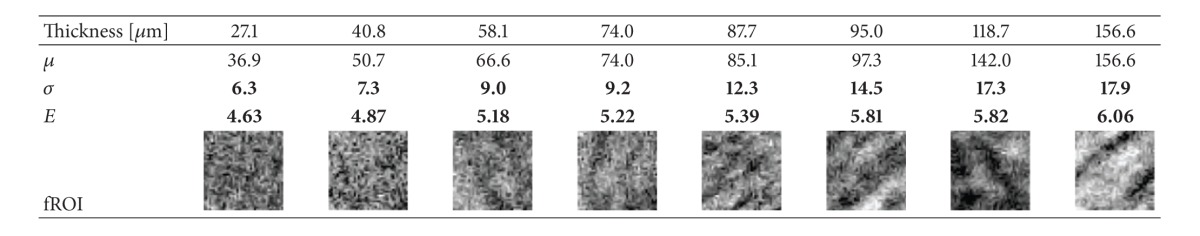# Analysis of Visual Appearance of Retinal Nerve Fibers in High Resolution Fundus Images: A Study on Normal Subjects

**DOI:** 10.1155/2013/134543

**Published:** 2013-12-29

**Authors:** Radim Kolar, Ralf P. Tornow, Robert Laemmer, Jan Odstrcilik, Markus A. Mayer, Jiri Gazarek, Jiri Jan, Tomas Kubena, Pavel Cernosek

**Affiliations:** ^1^Department of Biomedical Engineering, Faculty of Electrical Engineering and Communication, University of Technology, Technicka 12, 61600 Brno, Czech Republic; ^2^International Clinical Research Center, Center of Biomedical Engineering, St. Anne's University Hospital Brno, Pekarska 53, 65691 Brno, Czech Republic; ^3^Department of Ophthalmology, University of Erlangen-Nuremberg, Schwabachanlage 6, 91054 Erlangen, Germany; ^4^Pattern Recognition Lab and Erlangen Graduate School of Advanced Optical Technologies, University of Erlangen-Nuremberg, Martensstraße 3, 91058 Erlangen, Germany; ^5^Ophthalmology Clinic of Dr. Tomas Kubena, U Zimniho Stadionu 1759, 760 00 Zlin, Czech Republic

## Abstract

The retinal ganglion axons are an important part of the visual system, which can be directly observed by fundus camera. The layer they form together inside the retina is the retinal nerve fiber layer (RNFL). This paper describes results of a texture RNFL analysis in color fundus photographs and compares these results with quantitative measurement of RNFL thickness obtained from optical coherence tomography on normal subjects. It is shown that local mean value, standard deviation, and Shannon entropy extracted from the green and blue channel of fundus images are correlated with corresponding RNFL thickness. The linear correlation coefficients achieved values 0.694, 0.547, and 0.512 for respective features measured on 439 retinal positions in the peripapillary area from 23 eyes of 15 different normal subjects.

## 1. Introduction

The examination of the retina via an ophthalmoscope or fundus cameras (analog or digital) has been successfully used in diagnosis of many retinal and eye diseases [1]. Besides the optic disc, macula, and retinal vascular tree, the retinal nerve fiber layer (RNFL) can also be observed, particularly in a red-free light as proposed by Kulwant [2]. This layer creates a stripy-like texture pattern, which indicates the presence of nerve fibers. There has been an effort to analyze this layer in fundus images, which may improve the glaucoma diagnosis.  Table 1 summarizes several important papers, where RNF analysis in fundus photography (analog or digital) has been described using different approaches. One of the basic papers has been published in 1984 by Airaksinen et al. [3]. He described a method for RNFL quality evaluation around the optic disc using a scoring system. In 1996 the complex survey for visual RNFL analysis in fundus with respect to age and optic disc damage has been described by Jonas and Dichtl [4]. A simple texture analysis for severe RNFL defects detection has been described and tested by Yogesan at al., 1998 [5], on set of 10 digitized fundus photographs with low resolution. Tuulonen et al. [6] also described the microtexture analysis of RNFL in gray level digitized photographs. The local properties of texture based on brightness difference were computed and used as an input for classification between glaucoma and normal and ocular hypertension. In our former paper [7] we described the fractal based texture analysis method of RNFL and its application for classification of RNFL defects. Markov random field has been also used for similar purpose with simple and subjective comparison with the data from optical coherence tomography (OCT) [8] as well as directional spectral analysis and structural texture analysis [9]. An attempt for early glaucoma diagnosis is described in [10] where Gabor filters were used for detection of wider RNFL defects.

In spite of these applications it is still not clear what is the correlation between the parameters from the texture analysis and the RNFL thickness. Independent of texture analysis methods, the texture parameters (features) describe the texture visual appearance and they offer a tool for qualitative and semiquantitative inspection of RNFL thickness.

This paper describes the statistically based texture analysis of the RNFL in high resolution color fundus images of normal subjects and its correlation with RNFL thickness obtained by optical coherence tomography in the same subjects. The statistically based texture analysis makes the interpretation of the texture parameters well understandable and it is hypothesized that this analysis can be predictive and can lead to glaucoma diagnosis support. Although red-free photographs might be more appropriate for texture analysis, we have used color fundus images because they are widely distributed, inexpensive, and easy to acquire. In early glaucoma, the RNFL thinning preceded the optic disc damage and visual field loss so that RNFL can be used as a sensitive indicator of structural damage; see [17]. Recent papers, for example, [18], indicate that RNFL thickness measured by OCT can be used for diagnosis support in different stages of glaucoma [19], particularly in the early stage, where the RNFL thickness dramatically decreases.

**Figure 1:**
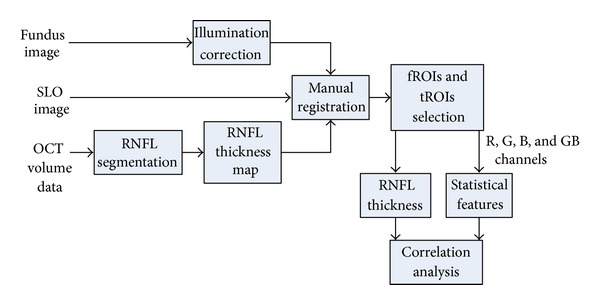
Flowchart of the proposed approach for RNFL visual appearance analysis. fROI stands for region of interest in fundus images and tROI stands for region of interest in RNFL thickness maps. See Section 2 for detailed description of each block.

The principle of the proposed method is shown in Figure 1 and this paper is organized as follows.  Section 2.1 shortly describes the acquisition devices and obtained images. Texture analysis of fundus image is described in Section 2.2 and RNFL segmentation in OCT B-scans in Section 2.3.  Section 2.4 describes the multimodal registration, which is needed for modality comparison. The results are discussed in Section 3 and the paper finishes with concluding remarks in Section 4.

## 2. Method

### 2.1. Data Acquisition

Color fundus images were taken by digital nonmydriatic fundus camera Canon CR-1 with a digital Canon camera EOS 40D (3888 × 2592 pixels, 45° field of view) on normal subjects without any suspected retinal or eye diseases. 23 color images (eyes) from 15 subjects taken on nondilated eyes in RAW (CR2) format were used for the presented analysis. Special care was taken during image acquisition—only sharp images were considered for presented analysis. For each analyzed eye, OCT volume scans were also acquired using a spectral domain OCT (Spectralis OCT, Heidelberg Engineering). Infrared reflection images (scanning laser ophthalmoscope, SLO) and OCT cross-sectional B-scan images of the dual laser scanning system were acquired simultaneously. From 61 to 121 B-scans per one eye were acquired, which corresponds to the spacing between each B-scan from 124.3 *μ*m to 63.1 *μ*m (30° field of view). An example of the positions of B-scans on the retinal surface is shown in Figure 3(a), where the SLO image, simultaneously acquired by OCT system, is also presented.

### 2.2. Texture Analysis of RNFL in Fundus Images

We have applied basic and advanced texture analysis methods in our previous work [7, 8, 20–22]. Statistical based methods are basic tool for the texture characterization and are also a promising tool for the RNFL texture analysis. There are three main classes of these methods: methods based on 1st-order statistics, 2nd-order statistics, and higher order statistics.

Here, we applied a first-order statistics, which depend only on the individual pixel value and not on the interaction between pixels. The main reason for this simple statistic is that the interpretation of these parameters is straightforward and gives a basic view on texture properties and its visual appearance. This statistic includes five parameters (features): mean, standard deviation, kurtosis, skewness, and Shannon entropy (as defined in information theory). They are calculated from intensity probability distribution, which must be estimated based on histogram of the analyzed image region. The definition and description of these parameters can be found elsewhere [23]. Here we present only the summarizing equations in Table 2.

The color fundus images were preprocessed in three steps. In the first step we reconstructed the RGB image from RAW data to TIFF format with linear gamma correction using DCRAW software [24]. This step is important, because we can achieve linear relation between image intensity and reflected intensity from retinal structures.

The second step is focused on removing the nonuniform illumination and increasing the contrast. Several methods were tested (e.g., [25, 26]) in order to increase the correlation between image features and RNFL thickness. Finally, the contrast limited adaptive histogram equalization (CLAHE) has been used [27]. This method locally enhances the contrast on small tiles, so that the histogram of output region has approximately uniform distribution. The size of tiles has been experimentally set to 20 × 20 pixels, but we observed that this size is not critical. The neighboring tiles are then interpolated to eliminate boundary artifacts. This approach has been applied on all color channels separately.

In the third step four grayscale images were generated for successive analysis. The red (R), green (G), and blue (B) channels were used separately. And finally the grayscale image computed as a mean of green and blue channels has been generated (GB image). The motivation for this step comes from the optical properties of green-blue filter, which is usually used for red-free fundus imaging. This green-blue channel combination also corresponds to absorption spectra of rhodopsin with maximum around 500 nm.

**Figure 2:**
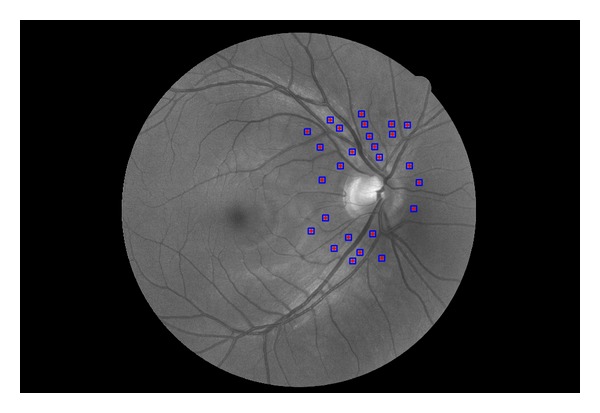
Fundus image (GB channel only) from our dataset with selected ROIs for analysis. These regions were manually placed apart from the blood vessels to not influence the texture features.

The data for the texture analysis was obtained by a manual selection of the small regions of interest (ROI) around the optic disc (Figure 2) including nasal, temporal, inferior, and superior area. The positions of ROIs correspond to various widths of the RNFL, given by the retinal physiology [28], to cover a large range of RNFL thickness. The size of ROI has been chosen to 41 × 41 pixels, which is a compromise between the ability to locally characterize texture by the features and the limitation to select sufficient number of these ROIs without blood vessels. These ROIs are located in close surroundings of the optic disc (approximately within the two optic disc diameters) and were carefully selected to exclude blood vessels and capillaries to remove their influence for the ROI texture analysis. The number of these ROIs in particular image is around 20 per each image. The total number of these ROIs for texture analysis is 439. These ROIs were defined in R, G, B, and GB channels and the above described statistical features were computed from each ROI. This leads to 20 features (5 features for each channel), which will be further analyzed.

One remark should be made here. Each subset of these samples comes from the same image, which implies their statistical dependence. Nevertheless, we can consider each ROI as representation of retinal structure at independent positions with various values of RNFL thickness and therefore these ROIs can be treated as statistically independent.

### 2.3. Segmentation in OCT Data

The OCT volume data has been processed in a semiautomatic way. In the first step, the inner limiting membrane (ILM) and the outer nerve fiber layer boundary (ONFL) have been automatically segmented. The parameters of the automated RNFL segmentation algorithm published in [29] have been adapted for the use on OCT volume scans. The algorithm can be summarized as follows. The retinal pigment epithelium (RPE) and ILM are detected by an edge detection taking the second derivative into account. After denoising the image with complex diffusion, the ONFL is found by an energy-minimization approach that takes the gradient as well as local and global smoothness constraints into account. The B-scans of the volume were segmented sequentially. This yielded segmentations that showed segmentation errors in a few cases, particularly in B-scans crossing the OD. In the second step, all segmentation errors were corrected manually using a nonparameterized curve (*free line*).

A Windows compiled version of the segmentation software can be downloaded under http://www5.informatik.uni-erlangen.de/research/software. It is called OCTSEG (optical coherence tomography segmentation and evaluation GUI) and may serve for many OCT related image processing purposes such as segmentation of the retinal layers and blood vessels and visualization of the results.

**Figure 3:**
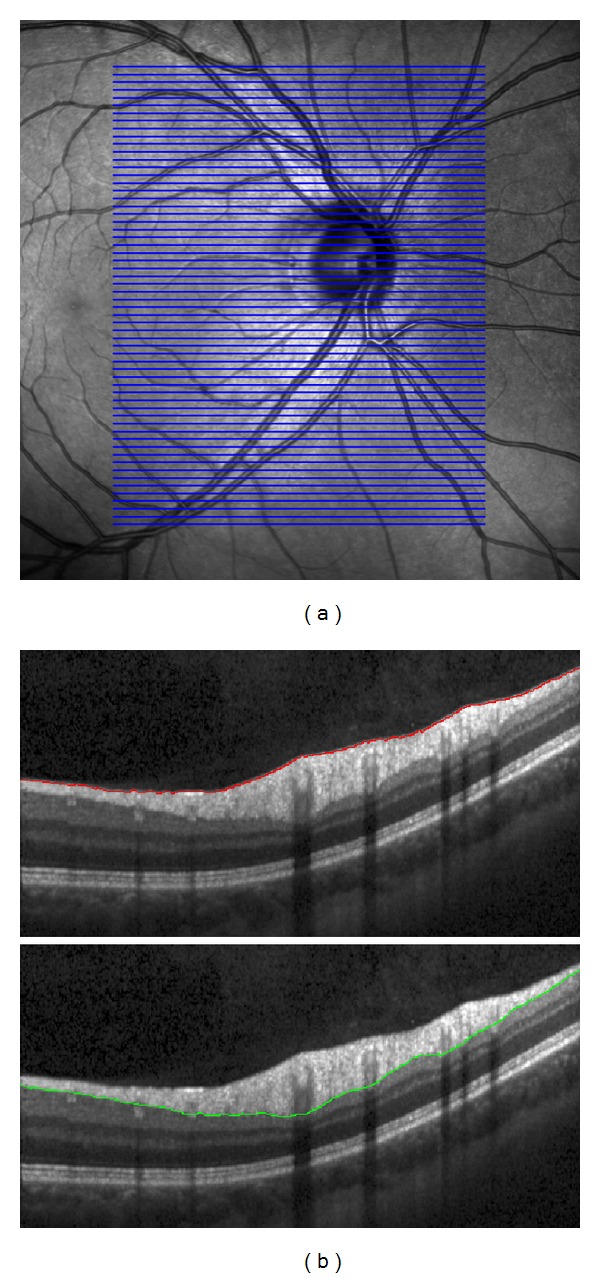
Spectralis SLO and OCT images. (a) SLO image. The blue lines represent the position of the B-scans on retinal surface (61 B-scans with spacing 124.3 *μ*m). (b) B-scan images with segmentation lines after manual correction, internal limiting membrane above and outer nerve fiber layer below.

**Figure 4:**
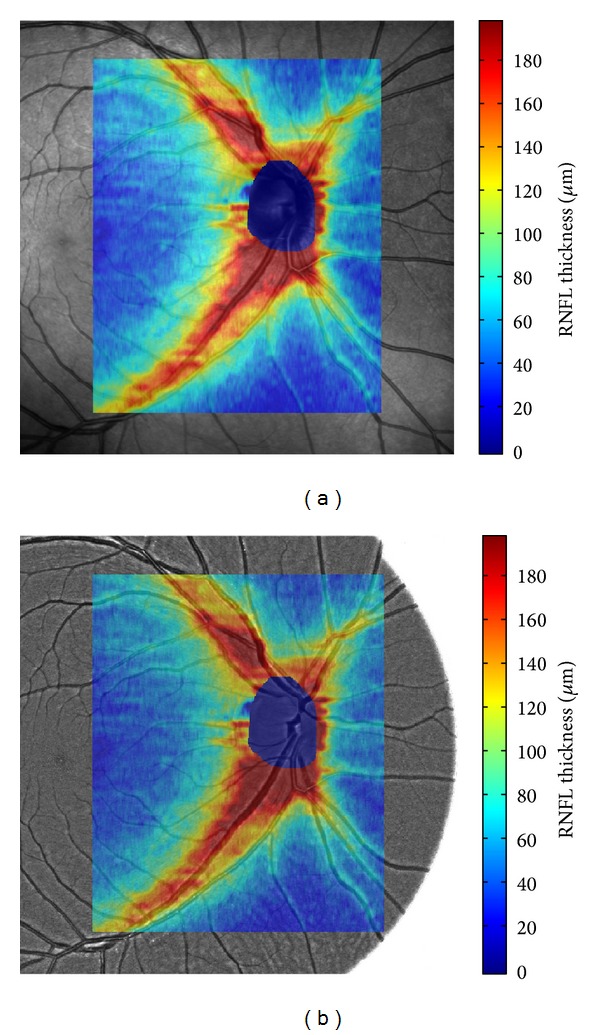
An example of the manually segmented RNFL mapped on the SLO image (a) and the green channel of fundus image (b). The colormap is scaled in *μ*m and the area around the optic disc has been removed because it does not contain the RNFL.

An example of the segmented ILM and ONFL is shown in Figure 3(b). This semiautomatic segmentation results in the RNFL thickness image, which is reconstructed from segmented B-scans. To ensure that the thickness image will have the same pixel size as the SLO image, an interpolation technique must be used (bilinear or spline interpolation is acceptable for our task [30]). Because we know the B-scans positions, we can map the thicknesses on the SLO image (see Figure 4(a)). This will be utilized in multimodal registration in the next section.

### 2.4. SLO to GB Image Registration

**Figure 5:**
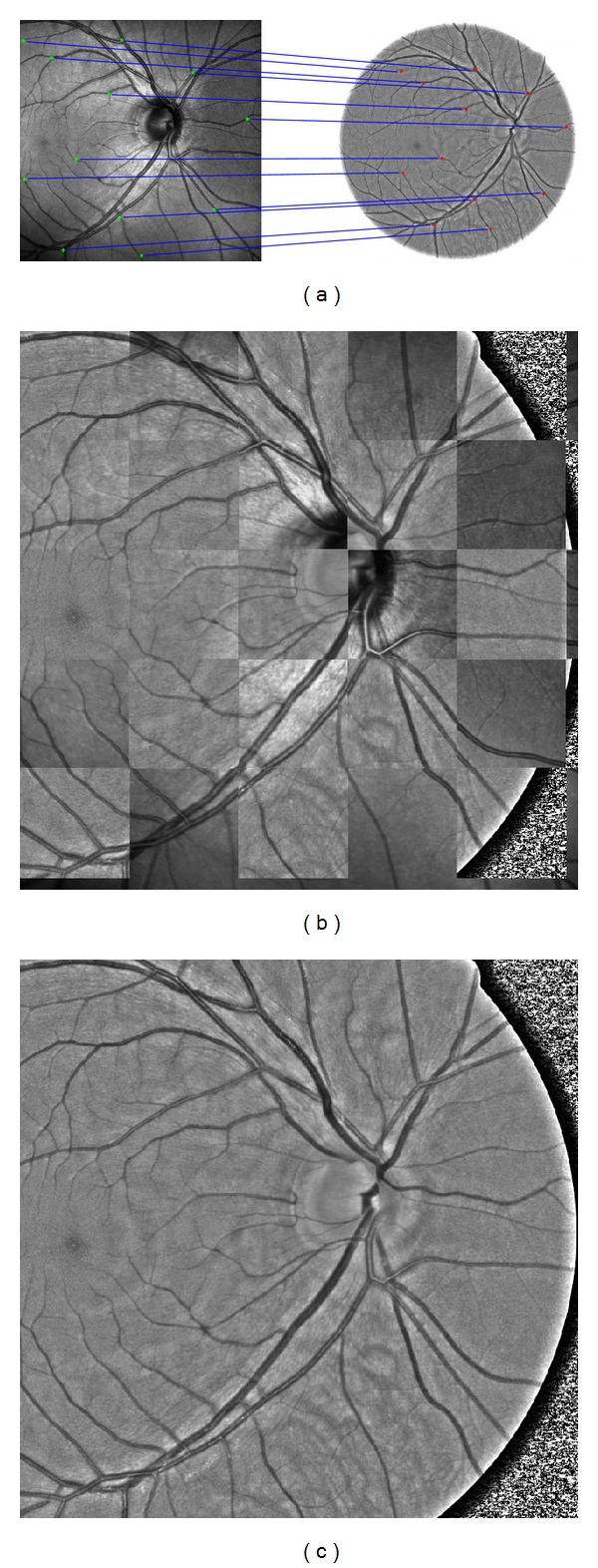
(a) Manually selected corresponding landmarks in SLO and fundus GB image. (b) Chessboard image from registered GB image. (c) Registered GB image.

To be able to compare the RNFL thickness map with the texture in the fundus images, image registration has to be performed. This bimodal registration (SLO to GB fundus image) can be automatic (e.g., [31, 32]) or manual. In this case we have used the registration based on manually selected landmarks positioned in the bifurcation points of the blood vessel tree. At least 12 landmarks were selected possibly uniformly throughout the images (Figure 5(a)). These are used for estimation of the spatial transformation parameters. Two kinds of spatial transformations are mostly used in retinal applications: affine and second-order polynomial transformations. Authors of [33] proved the validity of quadratic transformation model for curved retina, which is applicable particularly for images with a large field of view. We have also successfully tested this quadratic transformation together with the affine transformation, which gave us more precise results [34].

The 12-parametric second-order polynomial transformation model is described by [34]

(1) $$\begin{array}{c} \left(\begin{array}{c} x^{\prime }\\ y^{\prime } \end{array}\right)=\left(\begin{array}{cccccc} a_{11} & a_{12} & a_{13} & a_{14} & a_{15} & a_{16}\\ a_{21} & a_{22} & a_{23} & a_{24} & a_{25} & a_{26} \end{array}\right)\left(\begin{array}{c} x^{2}\\ xy\\ y^{2}\\ x\\ y\\ 1 \end{array}\right). \end{array}$$

Here, (*x*, *y*)^*T*^ denotes the coordinates of landmarks in a floating image (the image which will be aligned to the reference image) and (*x*′, *y*′)^*T*^ are the coordinates of these landmarks after transformation in a coordinate of the reference image. The image registration is defined as a minimization of sum of squared differences (*energy* function *ℰ*) between coordinates of corresponding landmarks in reference image (*X*, *Y*)^*T*^ and in transformed floating image (*x*′, *y*′)^*T*^:

(2) $$\begin{array}{c} ℰ=\overset{N}{\underset{i=1}{\sum }}{\left|\left(\begin{array}{c} x^{\prime }\\ y^{\prime } \end{array}\right)-\left(\begin{array}{c} X\\ Y \end{array}\right)\right|}^{2}\to \min, \end{array}$$

where *N* is a number of manually selected landmarks. Substitution leads to

(3) ℰ=∑i=1N(a11x2+a12xy+a13y2+a14x+a15y+a16−X)2+(a21x2+a22xy+a23y2+a24x+a25y+a26−Y)2.

The energy *ℰ* is minimized with the respect to entries of transformation matrix *a*_*ij*_. This leads to a set of linear equations, which can be easily solved by the Gauss elimination method [35]. An example of the registration result is shown in Figure 5 together with the manually selected landmarks and chessboard image. This processing has been applied on each image pair (SLO and GB images) in our dataset. This registration procedure enables an easy thickness image mapping on the fundus image. This is shown in Figure 4(b) together with SLO image. The next step is the analysis of the texture feature and RNFL thickness.

## 3. Results and Discussion

The result of so far described processing is a set of small ROIs in fundus images (fROI) and the corresponding ROIs in the thickness map (tROI). As mentioned, the size of fROI is 41 × 41 pixels, which has been chosen to span a sufficiently large region with RNFL striation. The maximum fROI size was limited by the blood vessels and other anatomical structures in the retinal image. From the tROI position (determined by the fROI position) the thickness has been estimated using the mean value from the 7 × 7 central window. This tROI size is equivalent to 0.0066 mm^2^.

### 3.1. Correlation Analysis

The first step of correlation analysis is focused on correlation between each feature and thickness. Spearman's rank correlation coefficients *R*_*S*_ have been calculated between each feature and corresponding RNFL thickness for each dataset of ROIs in each fundus image. The *R*_*S*_ values and basic statistics are summarized in Table 3. The correlation between R channel and thickness is the lowest for all R-channel features. The other channels have higher Spearman's correlation, particularly the features from GB channel (with *P* value < 0.05). Features computed from this channel are also better from the other point of view (low interimage *R*_*S*_ standard deviation and highest minimum and maximum correlations).

**Figure 6:**
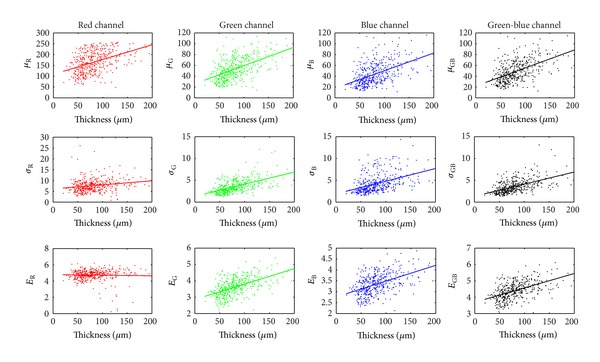
Scatter plots for three features (*μ*, *σ* and *E*) and RNFL thickness for different channels (R, G, B and GB).

The Spearman's correlation coefficients have been also computed between individual features and corresponding RNFL thickness considering the whole dataset of ROIs at once. These values are summarized in Table 4. The correlation value higher than 0.5 can be seen for most of the features from G, B, and GB channels. The scatter plots between features and thickness are shown in Figure 6. Rather high variance can be seen from this data. Nevertheless, the dependence of feature value on RNFL thickness is obvious. The linear fit is shown for illustration.

Each of the features from R has relatively low correlation (<0.5), which is probably caused by light reflections from the deeper retinal structures and therefore this channel is not convenient for RNFL texture analysis. Moreover, the light reflections within the red spectral band are relatively high and this reflected intensity can saturate the R channel of light sensor. These results indicate that the G, B, and GB channels are the most convenient channels for the texture analysis. It can be seen that the correlation coefficients of particular features are slightly higher for GB channel than single G and B channels. However, the correlation between particular features has also been investigated and it has been observed that there is a strong linear correlation between the same features computed from GB, G, or B channel (>0.86, *P*  value < 0.01), as can be expected. Therefore, we will use only the GB channel in further analysis. Another reason for GB channel priority is connected with fundus camera acquisition. It is clear that appearance of RNFL striation in G or B channels will depend on the properties of CMOS/CCD detection element in fundus camera. The combination of green and blue channels can decrease this dependence, because it combines the spectral characteristics of green and blue filters (which can be different for different manufacturers) and it is therefore more practical.

### 3.2. Regression Analysis

**Figure 7:**
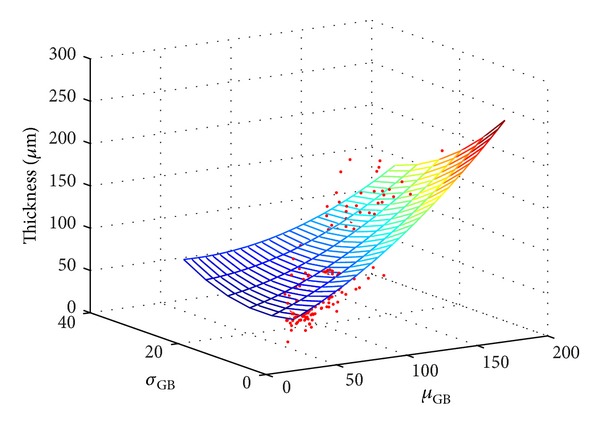
Graphical result of the multivariate regression analysis using the second-order polynomial model.

The multivariate nonlinear regression analysis has been applied to create a statistical model. The *μ*_GB_ and *σ*_GB_ values have been used as predictors and RNFL thickness as response. We used a second-order fitting model, which is appropriate considering the dependence of particular feature on thickness values, in the following form:

(4) $$\begin{array}{c} y=\beta_{1}+\beta_{2}\mu_{\text{GB}}+\beta_{3}\sigma_{\text{GB}}+\beta_{4}\mu_{\text{GB}}\sigma_{\text{GB}}+\beta_{5}\mu_{\text{GB}}^{2}+\beta_{6}\sigma_{\text{GB}}^{2}, \end{array}$$

where *β* is a vector of fitting coefficients. A nonlinear regression function *nlinfit* implemented in Matlab R2007b has been used. The results are graphically shown in Figure 7 and the estimated values are summarized in Table 5. The model was fitted on normalized data to be able to compare the influence of particular coefficients. One can see the highest linear dependence on *μ*_GB_. The *σ*_GB_ has similar influence for linear and quadratic terms.

This basic analysis shows that there is a correlation between several basic statistical features and the RNFL thickness measured quantitatively by OCT. An example of 8 selected fROIs (from GB channel) with corresponding feature values and RNFL thicknesses is shown in Table 6. It can be seen that with increasing RNFL thickness, the texture structure is changing from random to more organized. This is well described by the *μ*_GB_, *σ*_GB_, and *E*_GB_ values. The gray level mean value has straightforward interpretation—the reflected light intensity depends on the RNFL thickness. The standard deviation describes the “magnitude” of the gray level spatial variation of the nerve fibers independently from the light illumination. The Shannon entropy quantifies the shape of the intensity probability density function, estimated by histogram. More uniform histogram, which corresponds to area without RNFL, will have lower Shannon entropy value. On the other hand, stripy pattern due to RNFL will create higher peaks in histogram with higher Shannon entropy value. Skewness and kurtosis also describe the shape of the probability density function, but in different way, which is not significant in this case.

The regression model has been used to estimate the error of thickness estimation within each eye. The relative error of thickness estimation for each sample has been computed and the median value has been determined for each eye separately. This median value of errors ranges from 11.6% to 23.8% with mean value 16.9% and standard deviation 2.9%. The number of tested regions in retinal image ranges from 15 to 23. The level of this mean within-eye error and variance is promising, considering that we are using only two basic features: mean and variance texture features. The mean error also corresponds to MAE value of regression model for the whole datasets, which shows unbiased estimates of within-eye thicknesses. Nevertheless, it is expected that using more advanced texture analysis methods will enable creating more precise regression model.

## 4. Conclusion

This study on healthy subjects shows that basic local intensity analysis of the nerve fibers in the fundus photographs is related to RNFL thickness. The local reflected intensity in green-blue spectral band depends on RNFL thickness as well as the local standard deviation and Shannon entropy, which describe the probability density function of region intensities. The correlation between RNFL thickness and analyzed parameters is above 0.5. These values are mainly influenced also by the noise in fundus images, subjects variability, and also by inaccuracies in RNFL segmentation. However, we showed that when physicians analyze the fundus image, the local intensity variation on the nerve fiber branches is connected to RNFL thickness. A nonlinear statistical model has been built using the multivariate nonlinear regression with the mean absolute error 15.59 *μ*m. This model offers a possibility for raw estimation of RNFL thickness from texture features.

In conclusion two remarks should be emphasized. Only high quality and high resolution fundus images were used in this study. This is prerequisite for successful texture analysis. The second remark deals with RAW format. All images were acquired in RAW format and converted to lossless image format with linear gamma correction. If nonlinear gamma function is used, the feature values will result in a different dependence on RNFL thickness. This might influence the texture features and the visual appearance of RNFL thickness observed by physicians in fundus intensity image.

The texture analysis of the nerve fiber layer in fundus images seems to be a promising tool, which can be used for screening purposes and can be added as an additional feature to a fundus photography based screening protocol (e.g., the glaucoma risk index presented by Bock at al. [36]). The possibility and usefulness of automatic texture analysis in images of glaucoma patients will be investigated in a next step.

## Figures and Tables

**Table 1:** Short summarization of papers describing different approaches for the evaluation of RNF in fundus images (DCFI stands for digital colour fundus images).

Author	Method	Data	Results/description
Hoyt et al. (1973), [11]	The first subjective attempt to utilize fundus cameras for glaucoma detection by the evaluation of RNFL visual appearance. Comparison with perimetric findings.	A few number of black-and-white photographs	Funduscopic signs of the RNFL pattern provide the earliest objective evidence of nerve fiber layer atrophy in the retina.

Lundstrom and Eklundh (1980), [12]	Subjective visual evaluation of the changes in RNFL pattern intensity using fundus photographs.	A few number of black-and-white photographs	Findings that consecutive changes in RNFL pattern intensity are connected to progression of glaucoma disease.

Airaksinen et al. (1984), [3]	Subjective scoring of visual RNFL appearance in fundus photographs.	Black-and-white photographs (84 normals, 58 glaucomatous)	Confirmation of the dependence between changes in RNFL pattern and glaucoma progression in fundus photographs.

Peli (1988), [13]	Semiautomatic analysis of RNFL texture based on intensity information.	Digitized black-and-white photographs (5 normal, 5 glaucomatous, and 5 suspected of glaucoma)	Additional confirmation of the changes in RNFL intensity caused by glaucoma atrophy.

Yogesan et al. (1998), [5]	Automatic method for texture analysis of RNFL based on gray level run length matrices.	Digitized fundus photographs of size 648 × 560 pixels (5 normals, 5 glaucomatous)	Promising results for large focal wedge-shaped RNFL losses well outlined by surrounding healthy nerve fiber bundles. Diffuse RNFL loses could not be detected.

Tuulonen et al. (2000), [6]	Semiautomatic method using microtexture analysis of the RNFL pattern.	Digitized fundus photographs 1280 × 1024 pixels (7 normals, 9 glaucomatous, and 8 suspected of glaucoma	Showing that changes in a microtexture of RNFL pattern are related to glaucoma damage. There is a lack of small sample size.

Oliva et al. (2007), [14]	Semiautomatic method to texture analysis based on RNFL pattern intensity. Comparison with OCT measurement.	DCFI with size of 2256 × 2032 pixels (9 normals, 9 glaucomatous)	Correlation was only 0.424 between the intensity related parameters extracted from fundus images and RNFL thickness was measured by OCT.

Kolář and Jan (2008), [7]	Automatic method to texture analysis of RNFL based on fractal dimensions.	DCFI with size of 3504 × 2336 pixels (14 normal, 16 glaucomatous)	Local fractal coefficient was used as a feature for glaucomatous eye detection. There were problems with robust estimation of this coefficient.

Muramatsu, et al. (2010), [10]	Automatic approach with Gabor filters to enhance certain regions with RNFL pattern and clustering of these regions aimed to glaucoma detection.	DCFI with size of 768 × 768 pixels (81 normals, 81 glaucomatous)	The method is suitable only for detection of focal and wider RNFL losses expressed by significant changes in intensity.

Odstrcilik et al. (2010), [8]	Automatic method to texture analysis of RNFL based on Markov random fields.	DCFI with size of 3504 × 2336 pixels (18 normals, 10 glaucomatous)	The features ability to differentiate between healthy and glaucomatous cases is validated using OCT RNFL thickness measurement.

Prageeth et al. (2011), [15]	Automatic method to texture analysis using only intensity information about RNFL presence.	DCFI with size of 768 × 576 pixels (300 normals, 529 glaucomatous)	Intensity criteria were used. Detection of the substantial RNFL atrophy.

Acharya et al. (2011), [16]	Automatic analysis of RNFL texture using higher order spectra, run length, and cooccurrence matrices.	DCFI with size of 560 × 720 pixels (30 normals, 30 glaucomatous)	Specificity to detect glaucomatous eye is over 91%. The article does not explain thoroughly how the features were extracted and in which area of the image were computed.

Jan et al. (2012), [9]	Automatic method to RNFL texture analysis based on combination of intensity, edge representation, and Fourier spectral analysis.	DCFI with size of 3504 × 2336 pixels (8 normals, 4 glaucomatous)	The ability of proposed features to classify RNFL defects has been proven via comparison with OCT. The comparison was done only in a heuristic manner.

**Table 2:** Definitions of the first-order features used for analysis.

Mean	*μ* = ∑_*g*=0_^*G*−1^‍*gH*(*g*)	*H*(*g*) represents the probability density function, estimated from histogram *H*(*g*) = *n*_*g*_/*N*, where pixel value *g* = 0,1, 2,…, *G* − 1,*G* is a number of gray levels, *N* is a number of pixels in analyzed image, and *n*_*g*_ is a number of pixels with value *g*. *μ*_*n*_ represents statistical moment of *n*th order: *μ*_*n*_ = ∑_*g*=0_^*G*−1^‍(*g* − *μ*)^*n*^ *H*(*g*)
Standard deviation	*σ* = ∑_*g*=0_^*G*−1^‍(*g*−*μ*)^2^ *H*(*g*)	
Shannon entropy	*E* = −∑_*g*=0_^*G*−1^‍*H*(*g*)log⁡_2_(*H*(*g*))	
Skewness	*γ*_1_ = *μ*_3_/*μ*_2_^(3/2)^	
Kurtosis	*γ*_2_ = *μ*_4_/(*μ*_2_^2^ − 3)	

**Table 3:** The table summarizes the Spearman's correlation coefficients computed from samples in particular image. The mean value, standard deviation, and minimum and maximum values are presented together with mean *P* value. The described features (mean *μ*, standard deviation *σ*, and Shannon entropy *E*) were estimated in different channels (R, G, B, and GB).

Feature	*R*_*S*_ mean	*R*_*S*_ st. deviation	*R*_*S*_ min	*R*_*S*_ max	Mean *P* value
*μ*_R_	0.461	0.193	0.161	0.726	0.114
*σ*_R_	0.344	0.258	0.037	0.811	0.301
*E*_R_	0.212	0.249	−0.205	0.583	0.387

*μ*_G_	0.758	0.088	0.621	0.867	0.001
*σ*_G_	0.706	0.110	0.563	0.873	0.002
*E*_G_	0.646	0.104	0.492	0.830	0.006

*μ*_B_	0.750	0.116	0.516	0.874	0.003
*σ*_B_	0.702	0.107	0.549	0.872	0.002
*E*_B_	0.566	0.241	−0.015	0.848	0.110

*μ*_GB_	**0.765**	0.099	0.590	0.874	0.001
*σ*_GB_	**0.708**	0.108	0.559	0.869	0.002
*E*_GB_	**0.657**	0.096	0.531	0.844	0.004

**Table 4:** Spearman's correlation coefficients between considered features and RNFL thickness for the whole dataset; *P* value < 0.01.

*μ*_R_	*σ*_R_	*E*_R_	*μ*_G_	*σ*_G_	*E*_G_	*μ*_B_	*σ*_B_	*E*_B_	*μ*_GB_	*σ*_GB_	*E*_GB_
0.383	0.156	0.103	0.681	0.532	0.491	0.667	0.501	0.352	**0.694**	**0.547**	**0.512**

**Table 5:** The table shows the model coefficients, MAE (mean absolute error), and MCI (mean half width confidence interval).

*β*_1_	*β*_2_	*β*_3_	*β*_4_	*β*_5_	*β*_6_
80.53	24.40	−3.87	3.30	0.29	−3.41

MAE = 15.59, MCI = 4.44, *R* ^2^ = 0.531					

**Table 6:** Several selected fROIs are shown together with RNFL thickness and texture features computed from corresponding fROIs.